# Drivers of long‐term invertebrate community stability in changing Swedish lakes

**DOI:** 10.1111/gcb.14952

**Published:** 2020-01-14

**Authors:** Hannah B. Fried‐Petersen, Yimen G. Araya‐Ajoy, Martyn N. Futter, David G. Angeler

**Affiliations:** ^1^ Department of Aquatic Sciences and Assessment Swedish University of Agricultural Sciences Uppsala Sweden; ^2^ Centre for Biodiversity Dynamics Norwegian University of Science and Technology Trondheim Norway; ^3^ School of Natural Resources University of Nebraska – Lincoln Lincoln NE USA

**Keywords:** aquatic invertebrates, heterogeneous residuals, lakes, landscape ecology, spatial ecology, stability, time series

## Abstract

Research on ecosystem stability has had a strong focus on local systems. However, environmental change often occurs slowly at broad spatial scales, which requires regional‐level assessments of long‐term stability. In this study, we assess the stability of macroinvertebrate communities across 105 lakes in the Swedish “lakescape.” Using a hierarchical mixed‐model approach, we first evaluate the environmental pressures affecting invertebrate communities in two ecoregions (north, south) using a 23 year time series (1995–2017) and then examine how a set of environmental and physical variables affect the stability of these communities. Results show that lake latitude, size, total phosphorus and alkalinity affect community composition in northern and southern lakes. We find that lake stability is affected by species richness and lake size in both ecoregions and alkalinity and total phosphorus in northern lakes. There is large heterogeneity in the patterns of community stability of individual lakes, but relationships between that stability and environmental drivers begin to emerge when the lakescape, composed of many discrete lakes, is the focal unit of study. The results of this study highlight that broad‐scale comparisons in combination with long time series are essential to understand the effects of environmental change on the stability of lake communities in space and time.

## Graphical abstract

We assess the long‐term stability of invertebrate communities in 105 lakes in the Swedish “lakescape” by quantifying stability at the individual lake level as well as the environmental and physical variables affecting this stability at the ecoregion level. We find that stability is affected by species richness and lake size in both ecoregions, and alkalinity and total phosphorus in northern lakes. These results highlight the importance of broad‐scale and long‐term studies of stability.

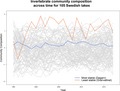

## 1 INTRODUCTION

Understanding the drivers of community stability through space and time is of key importance to manage and predict the effects of environmental change, especially in the face of increasing anthropogenic pressures (Hooper et al., 2005; Lewis & Maslin, 2015). Indeed, the relationship between community stability and disturbances has intrigued ecologists since at least the 1950s (McNaughton, 1977). However, despite a large body of theoretical and empirical studies about stability and resilience, landscape‐level or regional assessments are still rare (Hautier et al., 2014; Hector et al., 2010; Tilman, Reich, & Knops, 2006). Understanding broad‐scale ecological stability is necessary because many environmental pressures (species invasions, nutrient and acid deposition, climate change) operate at broad spatial and temporal scales and may cause long‐term loss of stability and resilience of entire landscapes (Allen et al., 2016; Angeler, Allen, Uden, & Johnson, 2015).

Community stability depends on both interactions among species and the sensitivity of each species to environmental fluctuations. Ecological stability is a multidimensional concept that describes different aspects of system dynamics and response to perturbations (Donohue et al., 2016). Pimm (1984) considered there to be five components of ecological stability: asymptotic stability, variability, persistence, resistance, and recovery (engineering resilience; Pimm, 1984). Variability, as the coefficient of variation over time, is a frequently used measure of stability; that is, stability as the inverse of variance (Ives, Klug, & Gross, 2000; Pimm, 1991). For the purposes of this paper, we are looking at variability as a component of stability and more specifically variability in species composition and abundance across time and space. The patterns of species distributions, abundances, and interactions may differ between spatial scales in an ecosystem and be driven by different processes (Leibold et al., 2004). Biotic communities are assembled from a combination of local and regional environmental factors, connectivity, and dispersal and the dynamics of metacommunities often differ from those of the local communities of which they are composed (Laliberté, Norton, & Scott, 2013; Leibold et al., 2004). Quantifying the stability of ecological communities at broad scales is a critical step in understanding, predicting, and managing consequences of environmental change such as biodiversity loss (Murphy & Romanuk, 2014) and spatial homogenization (Angeler, 2013; Dornelas et al., 2014).

Communities can become locally unstable in response to geographically restricted disturbances such as point source pollution or restricted habitat modification. Stability can also be measured at the landscape scale, in response to larger disturbances that affect many local communities within a landscape, such as air pollution and changing patterns of land use. It follows that local and regional community changes must not necessarily show the same patterns in response to changing environmental condition; that is, alpha (local) and gamma (regional) biodiversity and the turnover in community structure (beta) diversity do not need to follow the same patterns. For instance, biotic homogenization has been shown to occur at the landscape scale in response to eutrophication, despite local diversity being unchanged (Keith, Newton, Morecroft, Bealey, & Bullock, 2009). Additionally, invertebrate communities in Swedish lakes have become increasingly differentiated over time (“anti‐homogenization”) while nestedness, a concept related to species loss, has decreased (Angeler, 2013). This highlights the need to understand local patterns of stability and how this stability can change across the landscape scale.

Ecosystem stability and the link to biodiversity has also mainly been studied at the local scale (Delsol, Loreau, & Haegeman, 2018). Debate about the relationship between diversity and stability has a long history in ecology and is not yet settled but a consistent conceptual thread suggests that diversity will, on average, give rise to ecosystem stability (Loreau & de Mazancourt, 2013; McCann, 2000). The patterns of spatial scaling of biodiversity are well documented, particularly the species–area relationship which describes how species richness changes with area (Delsol et al., 2018). The spatial scaling of ecosystem stability and the link to biodiversity has received little attention. The maintenance of ecosystem structure and function requires an understanding of broader stability patterns at larger spatial scales that are more relevant for ecosystem management (Chalcraft, 2013; Peterson, Allen, & Holling, 1998).

We studied a 23 year time series of benthic invertebrates from 105 lakes across Sweden to quantify how stability changes between two ecoregions and how it relates to a large latitudinal gradient (from ~55° to 68°N), species richness, lake size, total phosphorus (TP), and alkalinity. We focus on invertebrate communities because they are sensitive to environmental change, a commonly used group in biomonitoring, and because they play key functional roles (e.g., leaf litter decomposition) in ecosystems (Bonada, Prat, Resh, & Statzner, 2006). We studied how changing abiotic conditions observed during the last decades have affected community composition. Abiotic change in Scandinavian freshwaters includes decreasing phosphorus concentrations (Huser, Futter, Wang, & Fölster, 2018) and changes in alkalinity due to acidification and subsequent recovery (Angeler & Drakare, 2013). Changes in the biotic environment have also been observed such as an increase in the distribution range of an invasive alga *Gonyostomum semen* (Angeler & Johnson, 2013) and changing patterns of biodiversity (Angeler & Drakare, 2013). These changes have been suggested as relevant for management and conservation (Angeler, 2013). It is imperative to assess how these changes not only affect lakes locally but also regionally.

**Figure 1:**
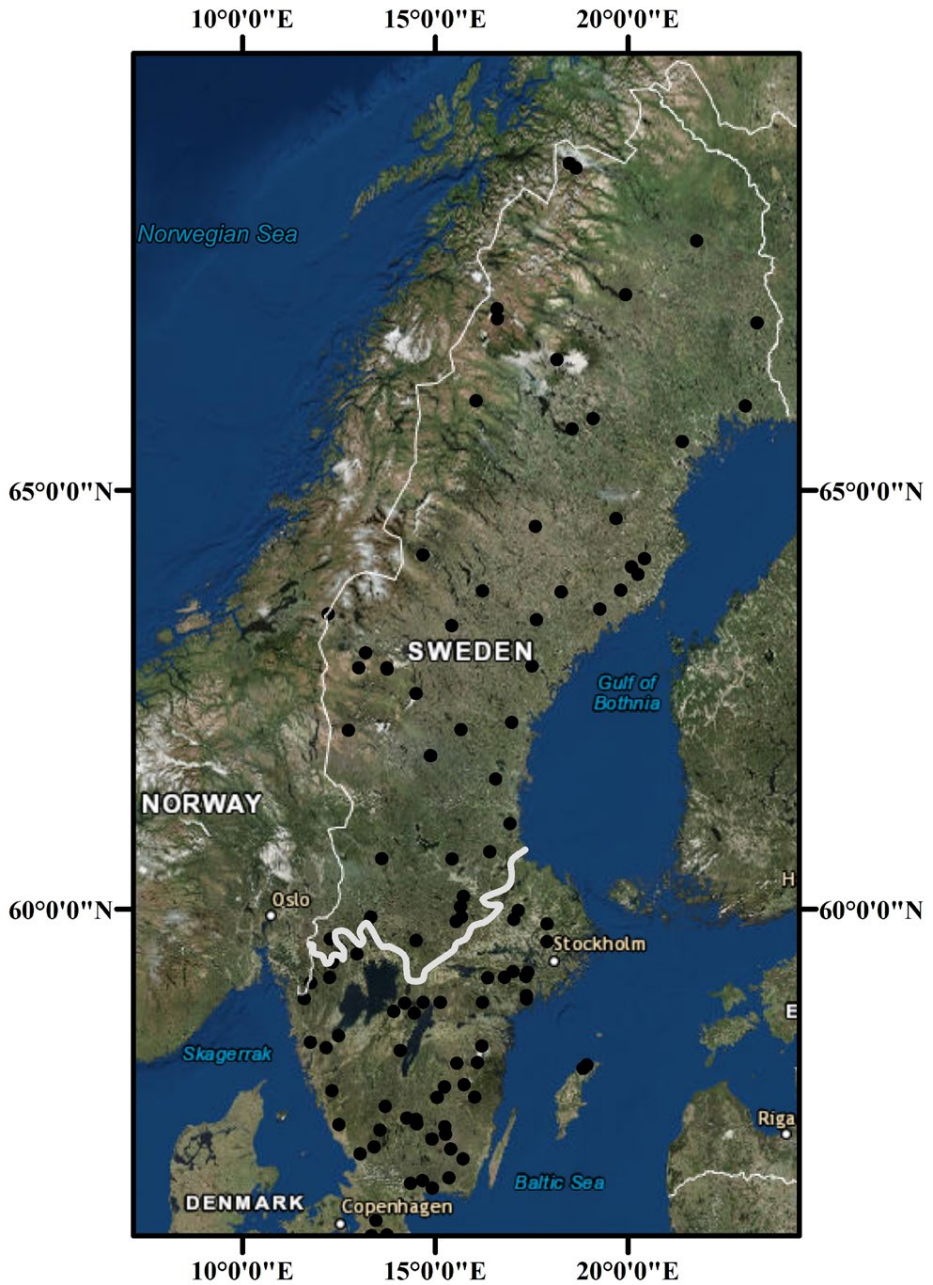
Map showing lake locations across Sweden below (*N* = 57) and above (*N* = 48) the Limes Norrlandicus

The studied lakes had minimal direct anthropogenic disturbance in the watershed (e.g., no point source pollution, urbanization, or agriculture) or to the lake itself (e.g., water regulation or stocking of fish). This allowed us to assess the effects of environmental pressures that are relevant at scales broader than an individual lake, and that may lead to cumulative or emergent properties at that scale, while accounting for relevant units of landscape structure which are required for an objective assessment of regional stability (Allen et al., 2016; McCluney et al., 2014; Sundstrom et al., 2017). For instance, ecoregions are spatial, often dynamic regions that are relatively homogeneous in terms of their ecological systems, organisms, environment, and anthropogenic effects (Roberts, Allen, Angeler, & Twidwell, 2019; Sandin & Johnson, 2000; Sundstrom et al., 2017). Indeed, aquatic ecosystems in different ecoregions (defined by more terrestrial features such as vegetation cover and land use) also often differ in their water quality and biota (Hughes & Larsen, 2002). Ecoregions can be a unit relevant for environmental management, and thus community stability at this scale is of interest. We used the “Limes Norrlandicus” (LN), which is a stable boundary in the Swedish landscape originally named by Carl Linnaeus (Oosthoek & Hölzl, 2018), to define two ecoregions in Sweden shown previously to differ in macroinvertebrate abundance and community structure: northern (above the LN) or southern (below LN) lakes (Sandin & Johnson, 2000; Figure 1). The LN is a strong biogeographical and climatic divide between northern and southern Sweden in terms of air temperature, precipitation (duration of snow cover), vegetation (e.g., boreal/alpine in the north vs. hemiboreal in the south) and soil type. Coinciding with different biotic structures, the pressures affecting the ecoregions above and below the LN are different due to higher human population density, more agriculture, higher storm intensity, and historically more acidification in the southern ecoregion. In contrast, the northern ecoregion shows a colder climate.

We assessed community composition and stability patterns of invertebrate communities at the ecoregion scale in Swedish lakes in three steps: (a) first, we summarized community composition for each lake in each year using detrended correspondence analysis (DCA: a unimodal multivariate ordination method); (b) we then examined the effects of different physical and environmental variables on community composition across two ecoregions (northern and southern Sweden); and (c) finally, we examined potential drivers of community stability at the ecoregion scale by analyzing the within‐lake variability in DCA scores across time and relating this to latitude, species richness, lake size, TP, and alkalinity.

## 2 MATERIALS AND METHODS

### 2.1 Study area

The Swedish National Lake Monitoring Program was developed in the 1960s and is unique in its temporal and spatial extent and open‐access policy (Fölster, Johnson, Futter, & Wilander, 2014). In 1995, lake littoral fauna were incorporated. The monitoring program is overseen and regulated by the Swedish Agency for Marine and Water Management (HaV: https://www.havochvatten.se/en). Data are open access and therefore no permission is required for their use (available in Swedish at: http://miljodata.slu.se/mvm/). For this study, fall sampling of environmental and littoral invertebrate community data from 105 lakes between 1995 and 2017 was used to cover lakes north and south of the Limes Norrlandicus (Figure 1). The studied lakes are medium sized (area = 0.03–14 km^2^, mean = 1.5 km^2^) and are considered within the monitoring program to be reference lakes, that is, the least disturbed in terms of no impact from point sources of pollution (Fölster et al., 2014). The sampling did not involve endangered or protected species.

### 2.2 Benthic invertebrate sampling

Sampling and analyses protocols for invertebrates and water chemistry were certified and quality controlled through the Swedish Board for Accreditation and Conformity Assessment (SWEDAC; http://www.swedac.se/en/). Sampling of benthic invertebrates followed Swedish standards (SS‐EN 27828) throughout the study period. Invertebrates were collected from each lake in one wind‐exposed, vegetation‐free littoral habitat during late autumn each year. In the most northern lakes, sampling was conducted at the end of September, so that similar seasonal conditions were covered during surveys. Five replicate samples were taken, using standardized kick sampling with a hand net (0.5 mm mesh size). For each sample, the bottom substratum was disturbed for 20 s along a 1 m stretch of the littoral zone at a depth of ~0.5 m. Invertebrate samples were preserved in 70% ethanol in the field and processed in the laboratory by sorting against a white background with 10× magnification. Invertebrates were identified to the finest taxonomic unit possible and counted using dissecting and light microscopes. Abundances are reported in the database as average number per sample, which is why there can be fractions of an individual. Taxa were identified according to a predetermined list of 517 operational taxonomic units, which were decided by expert opinion (Fölster & Wilander, 2007; Table S1).

### 2.3 Water chemistry sampling

Water quality data were obtained from surface water samples, which were taken at 0.5 m depth four to eight times each year at a mid‐lake station in each lake. Samples were collected with a Ruttner sampler and kept cool during transport to the laboratory, where they were analyzed for acidity (pH, alkalinity, SO_4_‐S concentration), nutrients (TP, total N, total organic C), and other variables. Total nitrogen was correlated with TP (*r* = .75, *p* < .05) in this study and was therefore excluded from analyses. All physicochemical analyses were conducted at the Department of Aquatic Sciences and Assessment (Swedish University of Agricultural Sciences) following international (ISO) or European (EN) standards (Wilander, Johnson, & Goedkoop, 2003). Autumn water chemistry measurements were matched by year and lake to the autumn invertebrate samples. Measurement intervals and analytical precision for each variable are available online at https://www.slu.se/en/departments/aquatic-sciences-assessment/laboratories/vattenlabb2/.

### 2.4 Analysis

#### 2.4.1 General procedure

We first summarized the community composition of the different lakes in each year using DCA. We then used linear mixed‐effects models to study the relationship between different variables and the DCA scores for each lake in each year. These models were then extended to estimate lake‐specific residual variation in their DCA scores across time as a measure of their (in)stability, which we will hereafter refer to as stability. We then proceeded to study the factors that predicted lake stability. All analyses were performed in R version 3.5.1 (R Development Core Team, 2018) and published data and code can be found in the Zenodo archive at http://doi.org/10.5281/zenodo.3384632.

#### 2.4.2 Summary of community composition

**Figure 2:**
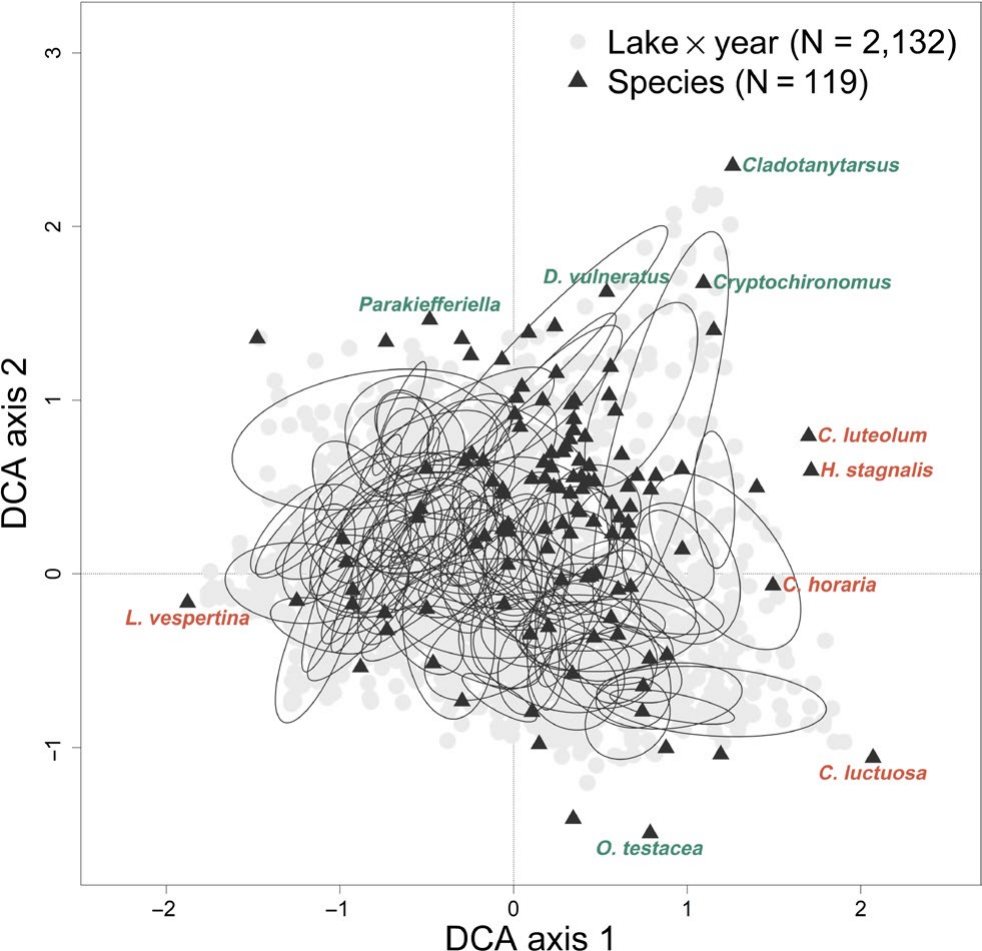
Detrended correspondence analysis ordination biplot for the 105 lakes across all years with rare species downweighted. Species locations in ordination space are depicted with black triangles and lake scores (in a given year) are depicted in gray circles. Black ellipses are drawn around the standard deviation of point scores for each lake, by year. Detrended correspondence analysis (DCA) axis 1 eigenvalue = 0.38 and axis length = 3.67. Axis 2 eigenvalue = 0.25 and axis length = 3.39. The five taxa with the highest absolute loadings for DCA 1 and DCA 2 are labeled in orange and green, respectively

To look at turnover and dissimilarity among the samples, we performed a DCA on raw littoral invertebrate abundance data. Multivariate ordination methods are appealing because they provide a robust estimate of community composition with large and noisy datasets. DCA represents assemblage samples as points in multidimensional space; similar assemblages are located close together and dissimilar assemblages further apart (Hill & Gauch, 1980). The detrending imposed by DCA has been criticized by some (see e.g., Borcard, Gillet, & Legendre, 2011; Wartenberg, Ferson, & Rohlf, 1987) and defended by others (e.g., ter Braak & Šmilauer, 2015), but we chose this method as it is well suited to ecological abundance data with long unimodal environmental gradients, and allows interpretations with biological relevance by examining the species that load most highly on the axes of interest (Palmer, 1993). We thus used DCA scores to quantify intra‐ and inter‐lake variability in taxonomic composition. The DCA was implemented using the function “decorana” in the vegan package (Oksanen et al., 2018). One DCA was performed for all 105 lakes across the study period (years 1995–2017, visualized in Figure 2), although not all lakes were sampled for all 23 years during the designated fall sampling period (minimum of 10 years, max of 23, and mean of ~20 years).

We screened the taxa list according to Angeler et al. (2015) by excluding taxa classified at higher taxonomic levels (i.e., family and above) to avoid unduly influencing results with classifications based on different hierarchical taxonomic levels. We also excluded taxa found in less than 5% of the samples and downweighted rare taxa to decrease the influence of extremely rare species on the DCA ordination, since we were particularly interested in overall changes in community composition across time. The species ranged in abundances in the dataset from 37.83 to 74,295.06 with a mean total abundance of 3,835.19 individuals (there can be fractions of an individual because abundance is reported as average number per sample). This resulted in an ordination with 2,132 lake‐year scores and 119 taxa scores (Figure 2). We do not use the eigenvalues as a measure of variance extracted because of the restrictions imposed by detrending, as suggested by McCune, Grace, and Urban (2002), but we considered that DCA axes should express the gradient lengths and summarize the community structure variation (ter Braak & Verdonschot, 1995). Compared with other ordination procedures, DCA has the advantage that the units are expressed in standard deviations of species turnover, which can be interpreted as turnover units of beta diversity. Variance along the first axis is often interpreted as proportional to the amount of species turnover among samples; that is, DCA gradient length provides an estimate of the amount of compositional change between samples (ter Braak, 1985). A long gradient usually has very few species shared by the sites at either end of the gradient. With gradient lengths greater than 4 *SD*, the data are expected to show clear unimodal (niche) structure, thus *SD* units of a DCA are a useful measure of beta diversity in the total dataset (ter Braak & Šmilauer, 2015). We performed further analyses on the lake scores (in both the northern and southern ecoregions) for the first two DCA axes, which summarize the most and second most variation in invertebrate community composition (eigenvalues and axes lengths for additional axes available in Table S2).

#### 2.4.3 Estimating environmental effects on DCA scores

We used linear mixed‐effects models to study the factors affecting the yearly DCA scores of the studied lakes following Equation (1). Lake yearly DCA scores along the first and second DCA ordination axis were modeled separately for each ecoregion, north and south of the Limes Norrlandicus. This resulted in a total of four models: DCA 1 south, DCA 2 south, DCA 1 north, and DCA 2 north. The models had as fixed effects the following variables: alkalinity, TP, and species richness. We also included lake size and latitude as fixed covariates. Size, TP, and alkalinity were log‐transformed because proportional changes in these variables are more biologically relevant than absolute changes. Latitude and richness were mean centered and standardized to the variable's standard deviation prior to analysis. These variables had correlations of 0.4 or less thus avoiding problems with multicollinearity. We also included random intercepts for lake and year identity in all models. It should be noted that alkalinity, richness, and TP can vary both within (across years) and between lakes while latitude and size can only vary between lakes. The parameterized mixed‐effects models can be described as:

(1) $$\begin{array}{cc} {\text{DCA score}}_{\mathrm{ij}} & =\beta_{0}+\beta_{1}\cdot {\text{lat}}_{i}+\beta_{2}\cdot {\text{size}}_{i}+\beta_{3}\cdot {\text{rich}}_{\mathrm{ij}}+\beta_{4}\cdot {\text{alk}}_{\mathrm{ij}}\\ & \;+\beta_{5}\cdot {\text{TP}}_{\mathrm{ij}}+u_{i}+v_{j}+\varepsilon_{\mathrm{ij}}, \end{array}$$

where DCA score*_ij_* is the DCA score (either 1 or 2, north or south) of a given lake (*i*) in a given year (*j*), *β*_0_ is the intercept, *β*_1_ through *β*_5_ are coefficients representing the effects of latitude, size, richness, alkalinity, and TP on DCA scores, respectively. *ε_ij_* reflects unmeasured effects on the DCA scores and were assumed to be normally distributed with a mean of zero and variance estimated from the data, *ε_ij_* ∼ *Ɲ* (0, V*_R_*). *u_i_* and *v_j_* specify random intercepts for lake (*i*) and year (*j*), that were also assumed to come from a normal distribution with means of zero and variances estimated from the data: *u_i_* ∼ *Ɲ* (0, V*_L_*) and *v_j_* ∼ *Ɲ* (0, V*_Y_*).

#### 2.4.4 Estimating lake stability

To examine larger regional patterns in stability, we first quantified stability at the lake level as the individual unit of measurement. We used the variation in yearly DCA scores within lakes and across years as a measure of individual lake stability. To estimate the within‐lake variation in yearly DCA scores, we extended the above‐mentioned mixed‐effects models to include lake‐specific “residual variation”, *ε_ij_* ∼ *Ɲ* (0, V*_Ri_*), where V*_Ri_* represents lake‐specific residual variation. This is equivalent to fitting a model with a heterogeneous residual structure as a function of lake identity (Gelman & Hill, 2007). The logarithm of the residual variance for each lake was also assumed to come from a normal distribution with variance estimated from the data. Thus, we obtained 105 within‐lake estimates of stability of yearly DCA scores and a measure of its variation (Table S3). These within‐lake variation estimates reflect the variation in yearly scores after accounting for the effects that the different variables may have on the DCA scores.

#### 2.4.5 Relating stability to latitude, size, richness, alkalinity, and TP

Ultimately, we were interested in examining larger regional patterns in lake stability as related to latitude, species richness, lake size, TP, and alkalinity. Thus as a next step, we examined how individual lake stability was related to gradients in the aforementioned variables. To this end, we extended the heterogenous residual models to include predictors for lake‐specific residual variances. Specifically, we modeled the lake‐specific variance in DCA scores as a function of latitude, lake mean richness across years, lake size, mean TP, and mean alkalinity. This was done by extending the previous models, but with the addition of modeling the lake‐specific residual variances as functions of lake latitude, size, and mean species richness, mean alkalinity, and mean TP, as shown in Equation (2).

(2) $$\begin{array}{cc} \log\left(V_{\mathrm{Ri}}\right) & =\beta_{0}+\beta_{1}\cdot {\text{lat}}_{i}+\beta_{2}\cdot {\text{size}}_{i}+\beta_{3}\cdot {\text{rich}}_{i}+\beta_{4}\cdot {\text{alk}}_{i}\\ & \;+\beta_{5}\cdot {\text{TP}}_{i}+e_{i}, \end{array}$$

where the log of lake‐specific residual variance is modeled as a function of latitude, size, and the mean lake values for richness, alkalinity, and TP.

#### 2.4.6 General modeling procedures and support for fixed and random effects

We fitted the mixed‐effects models described above using a Bayesian framework implemented in R version 3.4.2 (R Development Core Team, 2018) with the RJAGS package (Plummer, 2016). We ran 3,050,000 iterations per model, from which we discarded the initial 50,000 (burn‐in period). Each chain was sampled at an interval of 3,000 iterations, which resulted in a low autocorrelation among thinned samples. Posterior means and 95% credible intervals were estimated across the thinned samples for the mean effects (fixed effects), (co)variances, and heterogeneous residuals. Fixed‐effect priors were normally distributed and diffuse with a mean of zero and a large variance (100) and random‐effect priors were implemented as a positive uniform distribution with large variance (100).

We considered estimates of fixed effects and covariances to be significantly different from zero (i.e., in the frequentist's sense) when their associated 95% credible intervals did not overlap zero. We assessed the statistical support for a nonzero value of the heterogeneous residuals differently because variance components are bound to be positive and because prior choice can influence the credible intervals derived from the posterior distribution. We therefore determined the probability that an estimated variance was different from the null expectation based on permutation tests (Araya‐Ajoy & Dingemanse, 2017; Good, 1994). The DCA scores were randomly reallocated to different observations for each permutation. The resulting dataset had the same mean and variance as the observed dataset. This was done for all four datasets (DCA 1 and 2, above and below LN) and we then performed the four mixed‐effects models described above on the new datasets with randomized response variables, and estimated, for each permutation, a posterior mean value for each variance component of interest. This procedure was repeated 1,000 times to generate a “null” distribution of posterior mean estimates. We then calculated the probability (permutation.p) that the observed posterior mean value of a focal variance component was greater than any value expected from this permutation‐based null distribution. In this way, we could assess whether the variance in between‐lake stability was different from what is expected solely by chance and the data structure, that is, did lakes significantly differ in their stability.

## 3 RESULTS

### 3.1 Summary of community composition and residual variation in lake stability

Lakes varied considerably in their mean richness across years (minimum: 5 taxa, maximum: 47 taxa, mean: 29 taxa). Of the 119 taxa considered in the analyses, the five most common orders were Diptera (*N* = 40), Trichoptera (*N* = 29), Ephemeroptera (*N* = 9), Basommatophora (*N* = 9), and Coleoptera (*N* = 7). We summarized community composition within (across years) and between lakes using DCA on downweighted raw species abundances (Figure 2, Table S2). Since DCA axis 1 scales site scores in *SD* or turnover units of beta diversity, the length (3.67) means that sites at opposite ends of the gradients share very few taxa (i.e., there is a high beta diversity). DCA scores varied both between and within lakes, and it appears that within‐lake variation was different for the different lakes (Figure 2). The most important taxa associated with variation along DCA axis 1 belong largely to the order Ephemeroptera, although four other orders (Hirudinida, Diptera, Trichoptera, and Veneroida) loaded highly on this axis. Alternatively, DCA axis 2 was largely driven by taxa from the order Diptera (more specifically Chironomids, larvae of non‐biting midges), which load positively on DCA axis 2, that is, higher DCA 2 scores generally mean more Chironomids (Table 1).

**Table 1:** Top 15 taxonomic groups with the highest absolute scores on detrended correspondence analysis (DCA) axes 1 and 2

Taxon	Ord.	DCA 1 score	Abund.	Taxon	Ord.	DCA 2 score	Abund.
*Caenis luctuosa*	E	2.07	38,718	*Cladotanytarsus* sp.	D	2.35	19,649
*Leptophlebia vespertina*	E	−1.88	51,774	*Cryptochironomus* sp.	D	1.67	758
*Helobdella stagnalis*	H	1.71	781	*Demicryptochironomus vulneratus*	D	1.62	1,076
*Centroptilum luteolum*	E	1.7	3,652	*Oecetis testacea*	T	−1.49	454
*Caenis horaria*	E	1.49	29,687	*Parakiefferiella* sp.	D	1.46	4,967
*Psectrocladius* sp.	D	−1.48	31,179	*Dicranota* sp.	D	1.43	269
*Athripsodes* sp.	T	1.4	762	*Asellus aquaticus*	I	−1.41	74,295
*Cladotanytarsus* sp.	D	1.26	19,649	*Pisidium* sp.	V	1.4	21,595
*Agrypnia obsoleta*	T	−1.25	650	*Potthastia* sp.	D	1.39	345
*Tinodes waeneri*	T	1.19	1,390	*Psectrocladius* sp.	D	1.36	31,179
*Pisidium* sp.	V	1.15	21,595	*Tanytarsus* sp.	D	1.35	23,507
*Cryptochironomus* sp.	D	1.09	758	*Cricotopus* sp.	D	1.34	5,997
*Agrypnia* sp.	T	−0.98	510	*Cladopelma* sp.	D	1.26	1,201
*Orthotrichia* sp.	T	0.97	333	*Pseudosmittia* sp.	D	1.23	810
*Athripsodes cinereus*	T	0.97	543	*Molanna angustata*	T	1.19	387

Note

We also present their total abundances across all sites during the study period (1995–2017). Orders (Ord.) are as follows: D = Diptera, E = Ephemeroptera, H = Hirudinida, I = Isopoda, T = Trichoptera, V = Veneroida.

### 3.2 Estimating environmental effects on DCA scores

The community composition changed with lake latitude in both the ecoregions; in southern lakes only along DCA 2 and in northern lakes along both DCA 2 and DCA 1 (Table 2). Interestingly, the effect of latitude on DCA 2 score, which was largely driven by Chironomid taxa, was the opposite in southern and northern lakes, that is, more Chironomids in lower southern lakes and fewer Chironomids in lower northern lakes. Lake size, richness, alkalinity, and TP were also all important variables determining the structure of invertebrate community composition in the southern lakes. All relationships were positive along the DCA 1 so taxa positively associated with this axis (*Caenis luctuosa*, *Helobdella stagnalis*, *C. luteolum*, *C. horaria*) increased with these variables. Community composition in northern lakes was also affected by lake size, alkalinity, and TP (but not richness), and the relationships were also positive for DCA 1. Additionally, the random effects in all four models showed that there is substantial variation between lakes in community composition (Table 2).

**Table 2:** Results from the four mixed‐effects models used to study the drivers of community composition

Variable	DCA 1 South	DCA 2 South	DCA 1 North	DCA 2 North
Effect sizes (*β*)				
Intercept	−1.00 (−1.25, −0.75)	−1.43 (−1.73, −1.13)	−1.79 (−2.07, −1.49)	−0.96 (−1.22, −0.70)
Latitude	0.09 (−0.01, 0.19)	**−0.11** (−0.18, −0.04)	**−0.19** (−0.27, −0.12)	**0.13** (0.04, 0.22)
Size	**0.17** (0.09, 0.25)	−0.01 (−0.07, 0.04)	**0.06** (0, 0.12)	**−0.08** (−0.16, −0.01)
Richness	**0.02** (0, 0.04)	**−0.03** (−0.05, −0.01)	0.01 (−0.02, 0.03)	0.02 (−0.01, 0.05)
Alkalinity	**0.04** (0.02, 0.05)	0 (−0.01, 0.01)	**0.05** (0.02, 0.07)	**0.04** (0.01, 0.07)
Total phosphorus	**0.07** (0.01, 0.12)	0 (−0.04, 0.04)	**0.06** (0.02, 0.1)	−0.03 (−0.08, 0.03)
Variance estimates (*σ* ^2^)				
Lake	0.26 (0.19, 0.35)	0.12 (0.09, 0.17)	0.12 (0.09, 0.17)	0.16 (0.11, 0.22)
Year	0.00 (0.00, 0.01)	0.00 (0.00, 0.01)	0.00 (0.00, 0.00)	0.00 (0.00, 0.01)

Note

In this table, we present results of effects on detrended correspondence analysis (DCA) 1 and 2 scores, as measures of community composition. Models were performed separately for DCA axes 1 and 2 and for the two ecoregions (north and south). We present mean and 95% credible intervals for fixed and random effects. We also depict in bold those fixed‐effect estimates where the 95% credible intervals did not overlap zero.

### 3.3 Estimating residual variation in lake stability

We found significant differences between lakes in their stability across time in both the community structure captured by DCA 1 and DCA 2 in northern and southern lakes (permutation.*p* < .001 for all four models). These stability estimates were not affected by the differing temporal record for the lakes (model not shown). There was a slightly positive relationship between variation in DCA 1 and DCA 2 scores meaning that lakes that had large between‐year variation along DCA 1 also tended to have larger variation along DCA 2, though this relationship was only marginally significant (Pearson correlation *r* = .18, *p* = .07). The mean heterogeneous residual variance across lakes in DCA 1 score was 0.33 with a variance of 0.01, and the range spanned from 0.15 (Lake Dagarn, most stable) to 0.62 (Lake Granvattnet, least stable). For DCA 2, the mean heterogeneous residual variance was also 0.33 with a variance of 0.01, and a range from 0.13 (Siggeforasjön, most stable) to 0.93 (Ymsen, least stable). The values of stability for each lake are available in Table S3.

### 3.4 Relating stability to latitude, size, richness, alkalinity, and TP

**Figure 3:**
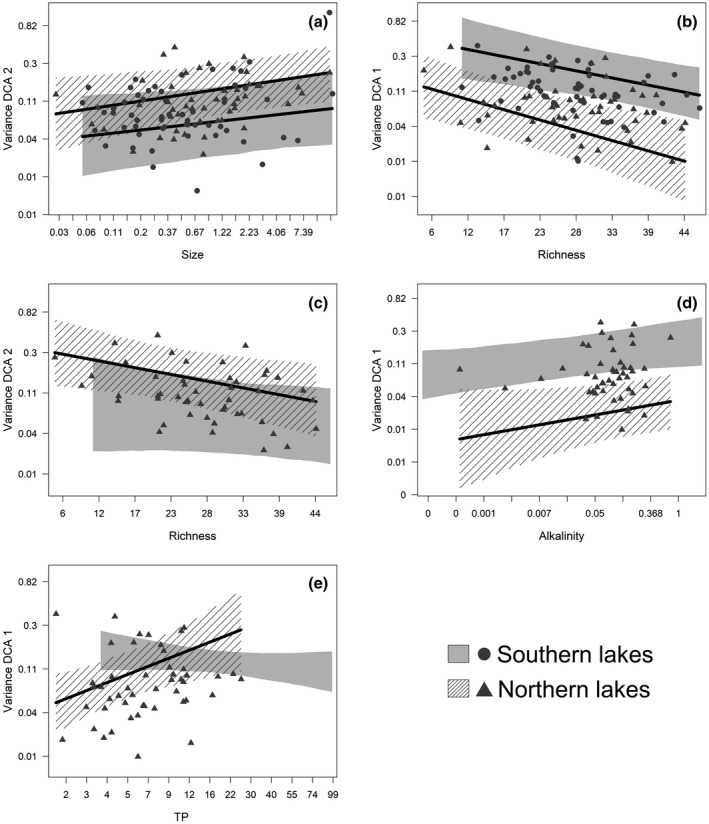
Modeled and observed effects on detrended correspondence analysis (DCA) 1 and DCA 2 scores for those variables with significant effects on community stability. Effects in northern and southern lakes are shown for comparison even if both were not significant. Lines indicate significant effects: (a) effect of size on DCA 2 score, (b) effect of richness on DCA 1 score, (c) effect of richness on DCA 2 score, (d) effect of alkalinity on DCA 1 score, and (e) effect of total phosphorus on DCA 1 score. Dots depict observed mean variation in raw DCA scores (filled circles for southern lakes, filled triangles for northern). The line is the model prediction of the heterogeneous residual variance in each lake after accounting for the fixed effects (with the 95% credible intervals in gray and shaded diagonal lines)

Community stability increased with richness in both northern and southern lakes for both DCA 1 and 2 scores; however, for DCA 2 in the southern lakes, the credible intervals slightly overlapped zero (Table 3). Larger lakes were also more unstable in the community composition captured by DCA 2, which may reflect lake size‐dependent variation in Chironomid communities. Northern lakes with higher levels of mean TP and mean alkalinity were less stable across time in the community composition captured by DCA 1 (Figure 3). Lake latitude did not affect community stability within either ecoregion.

**Table 3:** Results from the four mixed‐effects models used to estimate individual lake stability, and explore between‐lake patterns in stability

Variable	DCA 1 South	DCA 2 South	DCA 1 North	DCA 2 North
Effect sizes (*β*)				
Latitude	0.04 (−0.02, 0.1)	−0.08 (−0.15, 0)	−0.02 (−0.1, 0.06)	−0.06 (−0.14, 0.02)
Size	0 (−0.06, 0.05)	**0.08** (0.02, 0.14)	−0.01 (−0.08, 0.06)	**0.10** (0.03, 0.16)
Richness	**−0.15** (−0.23, −0.06)	−0.07 (−0.17, 0.02)	**−0.26** (−0.36, −0.15)	**−0.16** (−0.25, −0.06)
Alkalinity	0.02 (−0.06, 0.1)	−0.06 (−0.15, 0.03)	**0.09** (0, 0.17)	−0.01 (−0.08, 0.06)
Total phosphorus	−0.04 (−0.13, 0.04)	0.09 (−0.02, 0.19)	**0.27** (0.12, 0.42)	−0.07 (−0.19, 0.07)
Between‐lake variance in stability	0.07 (0.04, 0.10)	0.12 (0.08, 0.17)	0.09 (0.06, 0.14)	0.07 (0.04. 0.10)

Note

In this table, we present results of effects on the heterogeneous residual variances, used to study the drivers of community stability (inverse of variation in detrended correspondence analysis [DCA] scores). Models were performed separately for DCA axes 1 and 2 and for the two ecoregions (north and south). We present mean and 95% credible intervals. We also depict in bold those estimates where the 95% credible intervals did not overlap zero.

## 4 DISCUSSION

In this paper, we assessed broad‐scale composition and stability patterns of invertebrate communities in Swedish lakes. In a first tier of analysis, we summarized community composition for each lake in each year and assessed the impacts of broad environmental pressures and lake characteristics on invertebrate communities in the two ecoregions. We then quantified individual lake stability, and finally explored factors potentially influencing community stability in these two ecoregions. There was high beta diversity across the samples and the two DCA axes were driven by different invertebrate orders; taxa loading most highly on DCA 1 were largely Ephemeroptera, while DCA 2 was Chironomids from the order Diptera. Most of the included physical and chemical variables showed significant effects (of differing strengths and signs) on the DCA scores, which represented a summary of the invertebrate communities. We also found significant differences between lakes in their stability across this time series and explored the relevant variables affecting this stability. By quantifying lake stability across a spatially extensive area historically influenced by heterogeneous pressures on the aquatic environment, we could examine regional level, long‐term patterns in invertebrate community stability.

### 4.1 Drivers of community composition

The results from the DCA showed high β diversity across the lakes. The length of axis 1 means that few to no species were shared by communities at opposite ends, due to species turnover (*β*_turnover_ species replacement), or nestedness (*β*_nestedness_ richness differences between the samples; Soininen, Heino, & Wang, 2018). This is not surprising given the extensive latitudinal gradient between the south and north of Sweden as well as the different pressures suspected to affect the lake communities. Indeed, the strength and direction of some factors driving community composition differed between northern and southern lakes, in accordance with results found by Johnson (2000), which show differences in climate and vegetation between the two regions, and differences in invertebrate community structure between the middle and southern boreal regions, coinciding with the approximate position of the Limes Norrlandicus (LN). Our results support the notion that northern and southern lake regions comprise different spatial regimes, each with specific sets of structures and functions (Allen et al., 2016). This highlights the importance of accounting for the spatial structuring of bioclimatic regimes in order to avoid confounding results.

Invertebrate communities both north and south of the LN were influenced by latitude, although more strongly in the northern lakes (both DCA 1 and 2). This may reflect the larger geographic gradient in the northern ecoregion. Many variables may change with latitude including but not limited to: ice cover extent and duration, riparian vegetation, food web structure, temperature, and seasonal temperature cycle. Latitudinal effects may also reflect higher community turnover between lakes due to their larger geographic separation, which fits assumptions of distance‐decay models in ecology (Soininen, McDonald, & Hillebrand, 2007); that is, communities become more dissimilar in their composition the farther away their habitats are situated. A few previous studies from freshwater systems have found that species similarity between sites decays along spatial distances (Saito, Soininen, Fonseca‐Gessner, & Siqueira, 2015), especially if the spatial extent is large enough (Heino, 2011).

Alkalinity, lake size, and TP also affected community composition in southern and northern lakes. The effect of alkalinity fits with a large body of research showing acidification and subsequent recovery of Scandinavian freshwaters after the implementation of policy in the mid‐1980s, leading to a significant reduction in sulfur and nitrogen emissions to the atmosphere, and deposition to lakes (Futter, Valinia, Löfgren, Köhler, & Fölster, 2014; Skjelkvåle et al., 2005). While the chemistry of lakes in Sweden has begun to recover from acidification (Moldan, Cosby, & Wright, 2013), evidence of biological recovery has so far been equivocal (Angeler & Johnson, 2012). This may partly be due to lakes having been studied individually rather than collectively. Our analysis provides evidence that tolerance to acidic conditions is an important structuring force for invertebrate communities in Swedish lakes. That is, impact at the local scale of lakes can be highly variable and comprise a scale mismatch given that acidification impact is a regional rather than a local phenomenon. This means that despite acidification being a broad‐scale stressor, local conditions of lakes can mediate their degree of responses to this regional effect. Because disturbance impacts are strongly scale dependent (Nash et al., 2014), our results support the notion that impact is most accurately assessed when accounting for the appropriate scale at which stressors operate, that is, patterns may only manifest when studied between lakes and not within a single lake (Angeler, Allen, & Johnson, 2013). The observed effect of acidification on invertebrate communities is evident in some of the aforementioned taxa that load highly on DCA 1, which was related positively to alkalinity in both southern and northern lakes (Table 1). Loss of alkalinity in surface waters is an indicator of acidification (Futter et al., 2014) and important taxa like *Leptophlebia vespertina* (Ephemeroptera) have been shown to be highly tolerant to acidification, which is evidenced in the negative score along DCA 1 (smaller DCA 1 score means lower levels of alkalinity and more acidification). Conversely, *C. luctuosa* and *C. horaria* (Ephemeroptera) and *H. stagnalis* (Hirudinida) have been shown to be sensitive or highly sensitive to acidification (Schartau et al., 2008) and all have positive scores along DCA 1.

Lake size and TP as local measures were related to invertebrate community composition for lakes. A number of studies have shown the importance of lake size, measured as surface area and TP as important predictors of macroinvertebrate communities (Heino & Tolonen, 2017; Johnson, Goedkoop, & Sandin, 2004). The influence of size may be because of more complex and/or heterogeneous habitats in larger lakes (Heino, 2013).

### 4.2 Lake stability

We found differences in the stability of individual lakes. This allowed us to identify particularly stable and particularly variable lakes, which is of interest for management prioritization. Quantification of stability at the individual lake level allowed us to then look for patterns driving this stability at broader scales and between ecoregions. We expected that, as for the variables related to community composition, environmental variables such as alkalinity and TP, and physical variables like lake size and latitude along with species richness would drive patterns of stability across lakes. Clear broader scale patterns in stability emerged, namely that less alkaline, more species‐rich northern lakes with lower mean TP were more stable along DCA 1, as were smaller more species‐rich lakes along DCA 2. More species‐rich southern lakes were more stable along DCA 1 as were smaller lakes along DCA 2. We tried to avoid an overly large influence of extremely rare species on the DCA by removing them if they were present in less than 5% of the samples and downweighting the rare species within the DCA function, so the increased variance in more species poor lakes is likely not solely due to an analysis artifact.

Previous analyses of temporal patterns of biodiversity, which were based on a local scale of observation, have found that more diverse communities show smaller compositional changes over time, if most species weakly interact (McCann, Hastings, & Huxel, 1998; Yodzis, 1981). This may indicate that high diversity is associated with greater temporal stability in species composition (Shurin, 2007). Indeed, our results seem to support this relationship. The “insurance effect” has often been invoked to explain the positive relationship between richness and stability. This hypothesis posits that community level stability is dependent on the differential response of species or functional groups to varying conditions, as well as the functional redundancy of species that have important stabilizing roles (McCann, 2000). Disturbances may drive change in ecosystems by acting as a constraint for some species (i.e., stressor), while providing opportunity for others (i.e., resource), depending on their life history (Paine, Tegner, & Johnson, 1998). A major new insight gained from recent experimental work is that diversity may stabilize aggregate ecosystem or community properties while simultaneously destabilizing individual species abundances (Loreau & de Mazancourt, 2013). We acknowledge that while these explanations provide an important mechanistic understanding of community dynamics, our correlative study does not permit us to assess their relevance in our study.

We also found that northern lakes with higher mean TP tended to be less stable, which is interesting given recent studies of changing TP concentrations in Swedish lakes. TP tends to be declining across Swedish lakes and the largest relative declines are in northern Swedish lakes (Huser et al., 2018). Results from this analysis show that stability of invertebrate communities may be related to mean levels of TP, especially in the north.

### 4.3 Conclusion

Biodiversity and community stability at large scales (often termed gamma diversity) are not necessarily additive functions of biodiversity and community stability at smaller scales (alpha diversity; Vellend et al., 2013). Indeed at large spatial scales, community stability may be regulated by different mechanisms. For example, just as local diversity enhances the stability of local ecosystems, spatial compositional differences (β diversity) may reduce the variability of communities at regional scales (Aragón, Oesterheld, Irisarri, & Texeira, 2011; Pasari, Levi, Zavaleta, & Tilman, 2013). Despite advances in theoretical insight into community stability at broader spatial scales, a comprehensive framework has yet to be developed. Since management decisions often occur at the landscape scale, it is crucial to understand how stability manifests at the between‐lake level.

Our detected influence of ecoregion on stability suggests that accounting explicitly for spatial and/or biogeographical characteristics (location, connectivity, dispersal) may further shed light on regional‐scale patterns of stability, which need to be accounted for in management. Specifically, given that ecoregions differ in their environmental and biotic settings, a one‐size‐fits‐all management approach might not be efficient across ecoregions. Studies like ours provide managers with the necessary information to incorporate regional, rather than purely local lake conditions in their management schemes. A further benefit of regional approaches is that areas within a region where invertebrate communities are more susceptible to climate change can be managed in a spatially explicit way. That is, lakes may be identified that should receive management priority, which allows for a more targeted investment of management resources.

Accounting for regional patterns of stability captures more heterogeneity in the environment and differences between lakes. The relationships between species richness, TP, and stability become apparent only when we examine the lakescape level. Understanding regional ecological stability is important because environmental pressures are often not discriminating in their effects and as a result, larger spatial areas can be and often are affected. This study highlights the need for more spatially extensive studies of ecological community stability and the environmental variables affecting this stability. Studies of this nature and structure can be used to inform management about the effects of broad‐scale pressures that manifest at cross boundary levels, while still providing quantification of stability estimates at the individual lake level.

## Data Availability

The data and model codes that support the findings of this study are openly available and can be found in a Zenodo archive at http://doi.org/10.5281/zenodo.3384632.

## References

[gcb14952-bib-0001] Allen, C. R. , Angeler, D. G. , Cumming, G. S. , Folke, C. , Twidwell, D. , & Uden, D. R. (2016). Quantifying spatial resilience. Journal of Applied Ecology, 53(3), 625–635. 10.1111/1365-2664.12634

[gcb14952-bib-0002] Angeler, D. G. (2013). Revealing a conservation challenge through partitioned long‐term beta diversity: Increasing turnover and decreasing nestedness of boreal lake metacommunities. Diversity and Distributions, 19, 772–781. 10.1111/ddi.12029

[gcb14952-bib-0004] Angeler, D. G. , Allen, C. R. , & Johnson, R. K. (2013). Measuring the relative resilience of subarctic lakes to global change: Redundancies of functions within and across temporal scales. Journal of Applied Ecology, 50, 572–584. 10.1111/1365-2664.12092

[gcb14952-bib-0005] Angeler, D. G. , Allen, C. R. , Uden, D. R. , & Johnson, R. K. (2015). Spatial patterns and functional redundancies in a changing boreal lake landscape. Ecosystems, 18(5), 889–902. 10.1007/s10021-015-9871-z

[gcb14952-bib-0006] Angeler, D. G. , & Drakare, S. (2013). Tracing alpha, beta, and gamma diversity responses to environmental change in boreal lakes. Oecologia, 172(4), 1191–1202. 10.1007/s00442-012-2554-y 23229393

[gcb14952-bib-0008] Angeler, D. G. , & Johnson, R. K. (2012). Temporal scales and patterns of invertebrate biodiversity dynamics in boreal lakes recovering from acidification. Ecological Applications, 22, 1172–1186. 10.1890/11-1474.1 22827126

[gcb14952-bib-0009] Angeler, D. G. , & Johnson, R. K. (2013). Algal invasions, blooms and biodiversity in lakes: Accounting for habitat‐specific responses. Harmful Algae, 23, 60–69. 10.1016/j.hal.2013.01.001

[gcb14952-bib-0010] Aragón, R. , Oesterheld, M. , Irisarri, G. , & Texeira, M. (2011). Stability of ecosystem functioning and diversity of grasslands at the landscape scale. Landscape Ecology, 26(7), 1011–1022. 10.1007/s10980-011-9625-z

[gcb14952-bib-0011] Araya‐Ajoy, Y. G. , & Dingemanse, N. J. (2017). Repeatability, heritability, and age‐dependence of seasonal plasticity in aggressiveness in a wild passerine bird. Journal of Animal Ecology, 86(2), 227–238. 10.1111/1365-2656.12621 27973682

[gcb14952-bib-0012] Bonada, N. , Prat, N. , Resh, V. H. , & Statzner, B. (2006). Developments on aquatic insect biomonitoring: A comparative analysis of recent approaches. Annual Review of Entomology, 51(1), 495–523. 10.1146/annurev.ento.51.110104.151124 16332221

[gcb14952-bib-0013] Borcard, D. , Gillet, F. , & Legendre, P. (2011). Numerical ecology with R. New York, NY: Springer-Verlag 10.1007/978-3-319-71404-2

[gcb14952-bib-0014] Chalcraft, D. R. (2013). Changes in ecological stability across realistic biodiversity gradients depend on spatial scale. Global Ecology and Biogeography, 22(1), 19–28. 10.1111/j.1466-8238.2012.00779.x

[gcb14952-bib-0015] Delsol, R. , Loreau, M. , & Haegeman, B. (2018). The relationship between the spatial scaling of biodiversity and ecosystem stability. Global Ecology and Biogeography, 27(4), 439–449. 10.1111/geb.12706 29651225 PMC5892714

[gcb14952-bib-0016] Donohue, I. , Hillebrand, H. , Montoya, J. M. , Petchey, O. L. , Pimm, S. L. , Fowler, M. S. , … Yang, Q. (2016). Navigating the complexity of ecological stability. Ecology Letters, 19(9), 1172–1185. 10.1111/ele.12648 27432641

[gcb14952-bib-0017] Dornelas, M. , Gotelli, N. J. , McGill, B. , Shimadzu, H. , Moyes, F. , Sievers, C. , & Magurran, A. E. (2014). Assemblage time series reveal biodiversity change but not systematic loss. Science, 344(6181), 296–299. 10.1126/science.1248484 24744374

[gcb14952-bib-0018] Fölster, J. , Johnson, R. K. , Futter, M. N. , & Wilander, A. (2014). The Swedish monitoring of surface waters: 50 years of adaptive monitoring. Ambio, 43(S1), 3–18. 10.1007/s13280-014-0558-z 25403966 PMC4235935

[gcb14952-bib-0019] Fölster, J. , & Wilander, A. (2007). Sjöinventeringen 2005. En synoptisk vattenkemisk undersökning av Sveriges sjöar (Lake inventory 2005. A synoptic water chemistry survey of Sweden’s lakes). Rapport 2007:16.

[gcb14952-bib-0020] Futter, M. N. , Valinia, S. , Löfgren, S. , Köhler, S. J. , & Fölster, J. (2014). Long‐term trends in water chemistry of acid‐sensitive Swedish lakes show slow recovery from historic acidification. Ambio, 43(S1), 77–90. 10.1007/s13280-014-0563-2 25403971 PMC4235927

[gcb14952-bib-0021] Gelman, A. , & Hill, J. (2007). Data analysis using regression and multilevel/hierarchical models. Cambridge, UK: Cambridge University Press 10.1017/CBO9780511790942

[gcb14952-bib-0022] Good, P. (1994). Permutation tests. New York, NY: Springer New York 10.1007/978-1-4757-3235-1

[gcb14952-bib-0024] Hautier, Y. , Seabloom, E. W. , Borer, E. T. , Adler, P. B. , Harpole, W. S. , Hillebrand, H. , … Hector, A. (2014). Eutrophication weakens stabilizing effects of diversity in natural grasslands. Nature, 508(7497), 521–525. 10.1038/nature13014 24531763

[gcb14952-bib-0025] Hector, A. , Hautier, Y. , Saner, P. , Wacker, L. , Bagchi, R. , Joshi, J. , … Loreau, M. (2010). General stabilizing effects of plant diversity on grassland productivity through population asynchrony and overyielding. Ecology, 91(8), 2213–2220. 10.1890/09-1162.1 20836442

[gcb14952-bib-0026] Heino, J. (2011). A macroecological perspective of diversity patterns in the freshwater realm. Freshwater Biology, 56, 1703–1722. 10.1111/j.1365-2427.2011.02610.x

[gcb14952-bib-0027] Heino, J. (2013). Environmental heterogeneity, dispersal mode, and co‐occurrence in stream macroinvertebrates. Ecology and Evolution, 3, 344–355. 10.1002/ece3.470 23467653 PMC3586644

[gcb14952-bib-0028] Heino, J. , & Tolonen, K. T. (2017). Ecological drivers of multiple facets of beta diversity in a lentic macroinvertebrate metacommunity. Limnology and Oceanography, 62, 2431–2444. 10.1002/lno.10577

[gcb14952-bib-0029] Hill, M. O. , & Gauch, H. G. (1980). Detrended correspondence analysis: An improved ordination technique. Vegetatio, 42(1), 47–58. 10.1007/BF00048870

[gcb14952-bib-0030] Hooper, D. U. , Chapin, F. S. , Ewel, J. J. , Hector, A. , Inchausti, P. , Lavorel, S. , … Wardle, D. A. (2005). Effects of biodiversity on ecosystem functioning: A consensus of current knowledge. Ecological Monographs, 75(1), 3–35. 10.1890/04-0922

[gcb14952-bib-0031] Hughes, R. , & Larsen, D. P. (2002). Ecoregions: An approach to surface water protection. (JOURNAL VERSION). Washington, DC: U.S. Environmental Protection Agency. EPA/600/J‐88/224.

[gcb14952-bib-0032] Huser, B. J. , Futter, M. N. , Wang, R. , & Fölster, J. (2018). Persistent and widespread long‐term phosphorus declines in Boreal lakes in Sweden. Science of the Total Environment, 613–614, 240–249. 10.1016/j.scitotenv.2017.09.067 28915460

[gcb14952-bib-0033] Ives, A. R. , Klug, J. L. , & Gross, K. (2000). Stability and species richness in complex communities. Ecology Letters, 3(5), 399–411. 10.1046/j.1461-0248.2000.00144.x

[gcb14952-bib-0034] Johnson, R. K. (2000). Spatial congruence between ecoregions and littoral macroinvertebrate assemblages. Journal of the North American Benthological Society, 19(3), 475–486. 10.2307/1468108

[gcb14952-bib-0035] Johnson, R. K. , Goedkoop, W. , & Sandin, L. (2004). Spatial scale and ecological relationships between the macroinvertebrate communities of stony habitats of streams and lakes. Freshwater Biology, 49, 1179–1194. 10.1111/j.1365-2427.2004.01262.x

[gcb14952-bib-0036] Keith, S. A. , Newton, A. C. , Morecroft, M. D. , Bealey, C. E. , & Bullock, J. M. (2009). Taxonomic homogenization of woodland plant communities over 70 years. Proceedings of the Royal Society B, 276(1672), 3539–3544. 10.1098/rspb.2009.0938 19625318 PMC2817195

[gcb14952-bib-0037] Laliberté, E. , Norton, D. A. , & Scott, D. (2013). Contrasting effects of productivity and disturbance on plant functional diversity at local and metacommunity scales. Journal of Vegetation Science, 24(5), 834–842. 10.1111/jvs.12044

[gcb14952-bib-0038] Leibold, M. A. , Holyoak, M. , Mouquet, N. , Amarasekare, P. , Chase, J. M. , Hoopes, M. F. , … Gonzalez, A. (2004). The metacommunity concept: A framework for multi‐scale community ecology. Ecology Letters, 7(7), 601–613. 10.1111/j.1461-0248.2004.00608.x

[gcb14952-bib-0039] Lewis, S. L. , & Maslin, M. A. (2015). Defining the Anthropocene. Nature, 519(7542), 171–180. 10.1038/nature14258 25762280

[gcb14952-bib-0040] Loreau, M. , & de Mazancourt, C. (2013). Biodiversity and ecosystem stability: A synthesis of underlying mechanisms. Ecology Letters, 16, 106–115. 10.1111/ele.12073 23346947

[gcb14952-bib-0041] McCann, K. S. (2000). The diversity–stability debate. Nature, 405(6783), 228–233. 10.1038/35012234 10821283

[gcb14952-bib-0042] McCann, K. , Hastings, A. , & Huxel, G. R. (1998). Weak trophic interactions and the balance of nature. Nature, 395, 794 10.1038/27427

[gcb14952-bib-0043] McCluney, K. E. , Poff, N. L. R. , Palmer, M. A. , Thorp, J. H. , Poole, G. C. , Williams, B. S. , … Baron, J. S. (2014). Riverine macrosystems ecology: Sensitivity, resistance, and resilience of whole river basins with human alterations. Frontiers in Ecology and the Environment, 12, 48–58. 10.1890/120367

[gcb14952-bib-0044] McCune, B. , Grace, J. B. , & Urban, D. L. (2002). Analysis of ecological communities. Journal of Experimental Marine Biology and Ecology, 289(2), 303–305. 10.1016/S0022-0981(03)00091-1

[gcb14952-bib-0045] McNaughton, S. J. (1977). Diversity and stability of ecological communities: A comment on the role of empiricism in ecology. The American Naturalist, 111(979), 515–525. 10.1086/283181

[gcb14952-bib-0046] Moldan, F. , Cosby, B. J. , & Wright, R. F. (2013). Modeling past and future acidification of Swedish lakes. Ambio, 42(5), 577–586. 10.1007/s13280-012-0360-8 23288615 PMC3698327

[gcb14952-bib-0048] Murphy, G. E. P. , & Romanuk, T. N. (2014). A meta‐analysis of declines in local species richness from human disturbances. Ecology and Evolution, 4(1), 91–103. 10.1002/ece3.909 24455164 PMC3894891

[gcb14952-bib-0049] Nash, K. L. , Allen, C. R. , Angeler, D. G. , Barichievy, C. , Eason, T. , Garmestani, A. S. , … Sundstrom, S. M. (2014). Discontinuities, cross‐scale patterns, and the organization of ecosystems. Ecology, 95(3), 654–667. 10.1890/13-1315.1 24804450

[gcb14952-bib-0050] Oksanen, J. , Guillaume Blanchet, F. , Roeland Kindt, P. , Legendre, R. B. O'. H. , Simpson, G. L. , … Wagner, H. H. (2018). vegan: Community ecology package. R package version 2.5‐3.

[gcb14952-bib-0051] Oosthoek, K. J. , & Hölzl, R. (2018). Managing northern Europe’s forests: Histories from the age of improvement to the age of ecology. Berghahn Books, 10.1017/S0008938918000791

[gcb14952-bib-0052] Paine, R. T. , Tegner, M. J. , & Johnson, E. A. (1998). Compounded Perturbations Yield Ecological Surprises. Ecosystems, 1(6), 535–545. 10.1007/s100219900049

[gcb14952-bib-0053] Palmer, M. W. (1993). Putting things in even better order: The advantages of canonical correspondence analysis. Ecology, 74(8), 2215–2230. 10.2307/1939575

[gcb14952-bib-0054] Pasari, J. R. , Levi, T. , Zavaleta, E. S. , & Tilman, D. (2013). Several scales of biodiversity affect ecosystem multifunctionality. Proceedings of the National Academy of Sciences of the United States of America, 110(25), 10219–10222. 10.1073/pnas.1220333110 23733963 PMC3690867

[gcb14952-bib-0055] Peterson, G. , Allen, C. R. , & Holling, C. S. (1998). Ecological Resilience, Biodiversity, and Scale. Ecosystems, 1(1), 6–18. 10.1007/s100219900002

[gcb14952-bib-0056] Pimm, S. L. (1984). The complexity and stability of ecosystems. Nature, 307(5949), 321–326. 10.1038/307321a0

[gcb14952-bib-0057] Pimm, S. L. (1991). The balance of nature? : Ecological issues in the conservation of species and communities. University of Chicago Press. 10.1016/0169-5347(92)90160-D

[gcb14952-bib-0058] Plummer, M. (2016). rjags: Bayesian graphical models using MCMC. R Package Version 4‐6.

[gcb14952-bib-0059] R Core Team . (2018). R: A language and environment for statistical computing. Vienna, Austria: R Foundation for Statistical Computing Retrieved from https://www.R-project.org/

[gcb14952-bib-0060] Roberts, C. P. , Allen, C. R. , Angeler, D. G. , & Twidwell, D. (2019). Shifting avian spatial regimes in a changing climate. Nature Climate Change, 9(7), 562–566. 10.1038/s41558-019-0517-6

[gcb14952-bib-0061] Saito, V. S. , Soininen, J. , Fonseca‐Gessner, A. A. , & Siqueira, T. (2015). Dispersal traits drive the phylogenetic distance decay of similarity in Neotropical stream metacommunities. Journal of Biogeography, 42, 2101–2111. 10.1111/jbi.12577

[gcb14952-bib-0062] Sandin, L. , & Johnson, R. K. (2000). The statistical power of selected indicator metrics using macroinvertebrates for assessing acidification and eutrophication of running waters. Hydrobiologia, 422, 233–243. 10.1023/A:1017082619481

[gcb14952-bib-0063] Schartau, A. K. , Moe, S. J. , Sandin, L. , McFarland, B. , & Raddum, G. G. (2008). Macroinvertebrate indicators of lake acidification: Analysis of monitoring data from UK, Norway and Sweden. Aquatic Ecology, 42, 293–305. 10.1007/s10452-008-9186-7

[gcb14952-bib-0064] Shurin, J. B. (2007). How is diversity related to species turnover through time? Oikos, 116(6), 957–965. 10.1111/j.0030-1299.2007.15751.x

[gcb14952-bib-0065] Skjelkvåle, B. L. , Stoddard, J. L. , Jeffries, D. S. , Tørseth, K. , Høgåsen, T. , Bowman, J. , … Worsztynowicz, A. (2005). Regional scale evidence for improvements in surface water chemistry 1990–2001. Environmental Pollution, 137(1), 165–176. 10.1016/j.envpol.2004.12.023 15944047

[gcb14952-bib-0066] Soininen, J. , Heino, J. , & Wang, J. (2018). A meta‐analysis of nestedness and turnover components of beta diversity across organisms and ecosystems. Global Ecology and Biogeography, 27(1), 96–109. 10.1111/geb.12660

[gcb14952-bib-0067] Soininen, J. , McDonald, R. , & Hillebrand, H. (2007). The distance decay of similarity in ecological communities. Ecography, 30(1), 3–12. 10.1111/j.0906-7590.2007.04817.x

[gcb14952-bib-0069] Sundstrom, S. M. , Eason, T. , Nelson, R. J. , Angeler, D. G. , Barichievy, C. , Garmestani, A. S. , … Allen, C. R. (2017). Detecting spatial regimes in ecosystems. Ecology Letters, 20(1), 19–32. 10.1111/ele.12709 28000431 PMC6141036

[gcb14952-bib-0070] ter Braak, C. J. F. (1985). Correspondence analysis of incidence and abundance data: Properties in terms of a unimodal response model. Biometrics, 41, 859–873. 10.2307/2530959

[gcb14952-bib-0071] ter Braak, C. J. F. , & Šmilauer, P. (2015). Topics in constrained and unconstrained ordination. Plant Ecology, 216(5), 683–696. 10.1007/s11258-014-0356-5

[gcb14952-bib-0072] ter Braak, C. J. F. , & Verdonschot, P. F. M. (1995). Canonical correspondence analysis and related multivariate methods in aquatic ecology. Aquatic Sciences, 57(3), 255–289. 10.1007/BF00877430

[gcb14952-bib-0073] Tilman, D. , Reich, P. B. , & Knops, J. M. H. (2006). Biodiversity and ecosystem stability in a decade‐long grassland experiment. Nature, 441(7093), 629–632. 10.1038/nature04742 16738658

[gcb14952-bib-0074] Vellend, M. , Baeten, L. , Myers‐Smith, I. H. , Elmendorf, S. C. , Beausejour, R. , Brown, C. D. , … Wipf, S. (2013). Global meta‐analysis reveals no net change in local‐scale plant biodiversity over time. Proceedings of the National Academy of Sciences of the United States of America, 110(48), 19456–19459. 10.1073/pnas.1312779110 24167259 PMC3845118

[gcb14952-bib-0075] Wartenberg, D. , Ferson, S. , & Rohlf, F. J. (1987). Putting things in order: A critique of detrended correspondence analysis. The American Naturalist, 129(3), 434–448. 10.1086/284647

[gcb14952-bib-0076] Wilander, A. , Johnson, R. K. , & Goedkoop, W. (2003). Riksinventering 2000: En synoptisk studie av vattenkemi och bottenfauna i Svenska sjöar och vattendrag. Department of Environmental Assessment, Swedish University of Agricultural Sciences. Report 2003 (1), 117.

[gcb14952-bib-0077] Yodzis, P. (1981). The stability of real ecosystems. Nature, 289, 674 10.1038/289674a0

