# Supplementary material for: Drivers of long‐term invertebrate community stability in changing Swedish lakes
Source: Glob Chang Biol. 2020 Jan 14;26(3):1259–70. doi: 10.1111/gcb.14952 (PMC7078863; doi:10.1111/gcb.14952)
Supplement: Supplementary file 1 [file GCB-26-1259-s001.docx]

**Supplementary materials**

**Table S1**. Full list of the 119 taxa used in the analyses. This list of taxa was obtained by selecting those taxa found in more than 5% of the samples from a larger list screened by Angeler et al. 2015 and by excluding taxa classified at higher taxonomic levels (that is, family and above) to avoid unduly influencing results with ambiguous classifications.

| **Taxa** |  |  |
| --- | --- | --- |
| *Ablabesmyia longistyla* | *Endochironomus* sp. | *Nemoura* sp. |
| *Ablabesmyia monilis* | *Ephemera vulgata* | *Nemoura avicularis* |
| *Ablabesmyia phatta* | *Epoicocladius ephemerae* | *Nemoura cinerea* |
| *Acroloxus lacustris* | *Erpobdella octoculata* | *Oecetis* sp. |
| *Aeshna grandis* | *Erythromma najas* | *Oecetis testacea* |
| *Agraylea* sp. | *Gammarus pulex* | *Orectochilus villosus* |
| *Agrypnia* sp*.* | *Glossiphonia* sp. | *Orthotrichia* sp. |
| *Agrypnia obsoleta* | *Glossiphonia complanata* | *Oulimnius tuberculatus* |
| *Ameletus inopinatus* | *Glyptotendipes* sp*.* | *Oxyethira* sp. |
| *Argyroneta aquatica* | *Gyraulus acronicus* | *Pagastiella orophila* |
| *Asellus aquaticus* | *Gyraulus albus* | *Parachironomus* sp. |
| *Athripsodes* sp. | *Gyraulus crista* | *Parakiefferiella* sp*.* |
| *Athripsodes aterrimus* | *Gyrinus* sp. | *Paramerina* sp. |
| *Athripsodes cinereus* | *Haliplus* sp. | *Paratanytarsus* sp. |
| *Bathyomphalus contortus* | *Helobdella stagnalis* | *Phaenopsectra* sp*.* |
| *Bithynia tentaculata* | *Heterotanytarsus apicalis* | *Phryganea bipunctata* |
| *Caenis horaria* | *Heterotrissocladius grimshawi* | *Phryganea grandis* |
| *Caenis luctuosa* | *Heterotrissocladius marcidus* | *Pisidium* sp. |
| *Capnia* sp. | *Hippeutis complanatus* | *Polycentropus flavomaculatus* |
| *Centroptilum luteolum* | *Holocentropus* sp. | *Polycentropus irroratus* |
| *Chaoborus flavicans* | *Holocentropus dubius* | *Polypedilum* sp. |
| *Cladopelma* sp. | *Hydracarina* sp. | *Potthastia* sp. |
| *Cladotanytarsus* sp. | *Hydroptila* sp. | *Procladius* sp*.* |
| *Clinotanypus nervosus* | *Hygrotus* sp. | *Psectrocladius* sp. |
| *Cloeon* sp. | *Ilybius* sp*.* | *Pseudochironomus prasinatus* |
| *Coenagrion* sp. | *Kageronia fuscogrisea* | *Pseudosmittia* sp. |
| *Conchapelopia* sp. | *Lauterborniella agrayloides* | *Radix* sp. |
| *Cordulia aenea* | *Lepidostoma hirtum* | *Radix balthica* |
| *Corynoneura* sp. | *Leptophlebia marginata* | *Sialis lutaria* |
| *Cricotopus* sp. | *Leptophlebia vespertina* | *Sigara* sp. |
| *Cryptochironomus* sp. | *Limnephilus* sp. | *Somatochlora metallica* |
| *Cyrnus flavidus* | *Micronecta* sp. | *Stempellina* sp. |
| *Cyrnus insolutus* | *Microtendipes pedellus* | *Stenochironomus* sp. |
| *Cyrnus trimaculatus* | *Molanna angustata* | *Stictochironomus* sp. |
| *Demicryptochironomus vulneratus* | *Molannodes tinctus* | *Synorthocladius semivirens* |
| *Dendrocoelum lacteum* | *Mystacides* sp. | *Tanytarsus* sp. |
| *Dicranota* sp. | *Mystacides azurea* | *Tinodes waeneri* |
| *Dicrotendipes* sp. | *Nebrioporus depressus* | *Tribelos* sp. |
| *Diura nanseni* | *Nematoda* sp. | *Turbellaria* sp. |
| *Ecnomus tenellus* | *Nemotaulius punctatolineatus* |  |

**Table S2.** Eigenvalues and axis lengths for all four DCA axes.

|  | **DCA 1** | **DCA 2** | **DCA 3** | **DCA 4** |
| --- | --- | --- | --- | --- |
| **Eigenvalues** | 0.383 | 0.2463 | 0.1921 | 0.1984 |
| **Axis lengths** | 3.6645 | 3.3906 | 3.159 | 3.0702 |

**Table S3**. Residual variation in lake DCA scores across years after accounting for variance explained by fixed and random effects (heterogeneous residuals). Variance is sorted in ascending order by DCA 1 score so Dagarn is the most stable, least variable lake while Granvattnet is the most variable, least stable lake in terms of DCA 1 score. 95% credible intervals are reported in parentheses. Location north or south of the Limes Norrlandicus is noted in the last column.

| **Lake** | **DCA 1** | **DCA 2** | **LN** |
| --- | --- | --- | --- |
| Dagarn | 0.02 (0.01, 0.04) | 0.1 (0.07, 0.15) | North |
| Louvvajaure | 0.03 (0.02, 0.05) | 0.06 (0.04, 0.09) | North |
| Vitträsket | 0.03 (0.02, 0.06) | 0.08 (0.05, 0.14) | North |
| Krankesjön | 0.03 (0.02, 0.06) | 0.12 (0.08, 0.19) | South |
| Limmingsjön | 0.04 (0.02, 0.05) | 0.11 (0.07, 0.16) | North |
| Båtkåjaure | 0.04 (0.02, 0.06) | 0.11 (0.06, 0.19) | North |
| Tväringen | 0.04 (0.02, 0.06) | 0.1 (0.07, 0.15) | North |
| Fagertärn | 0.04 (0.02, 0.06) | 0.03 (0.02, 0.05) | South |
| Östra Helgtjärnen | 0.04 (0.02, 0.07) | 0.08 (0.05, 0.14) | North |
| Ögerträsket | 0.05 (0.02, 0.09) | 0.07 (0.04, 0.12) | North |
| Stor-Arasjön | 0.05 (0.03, 0.07) | 0.14 (0.09, 0.2) | North |
| Hällsjön | 0.05 (0.03, 0.08) | 0.15 (0.1, 0.21) | North |
| Stensjön | 0.05 (0.03, 0.08) | 0.06 (0.04, 0.09) | North |
| Fiolen | 0.05 (0.03, 0.08) | 0.23 (0.15, 0.33) | South |
| Fräcksjön | 0.05 (0.03, 0.08) | 0.03 (0.02, 0.05) | South |
| Grissjön | 0.05 (0.03, 0.08) | 0.12 (0.08, 0.17) | South |
| Norra Reivo | 0.05 (0.03, 0.09) | 0.09 (0.05, 0.15) | North |
| Valkeajärvi | 0.05 (0.03, 0.09) | 0.11 (0.07, 0.16) | North |
| Vuolejaure | 0.05 (0.03, 0.09) | 0.09 (0.05, 0.16) | North |
| Hällvattnet | 0.05 (0.04, 0.08) | 0.16 (0.11, 0.23) | North |
| Skärsjön | 0.05 (0.04, 0.08) | 0.03 (0.02, 0.04) | South |
| Rotehogstjärnen | 0.06 (0.04, 0.08) | 0.08 (0.05, 0.13) | South |
| Spjutsjön | 0.06 (0.04, 0.09) | 0.13 (0.09, 0.18) | North |
| Täftesträsket | 0.06 (0.04, 0.09) | 0.14 (0.1, 0.2) | North |
| Rammsjön | 0.06 (0.04, 0.09) | 0.06 (0.04, 0.1) | South |
| Gipsjön | 0.06 (0.04, 0.1) | 0.07 (0.05, 0.11) | North |
| Björken | 0.06 (0.04, 0.1) | 0.05 (0.04, 0.08) | South |
| Skärgölen | 0.07 (0.04, 0.09) | 0.05 (0.03, 0.07) | South |
| Långsjön | 0.07 (0.04, 0.1) | 0.06 (0.04, 0.09) | North |
| Abiskojaure | 0.07 (0.04, 0.11) | 0.2 (0.13, 0.29) | North |
| Stor-Tjulträsket | 0.07 (0.04, 0.11) | 0.14 (0.09, 0.21) | North |
| Edasjön | 0.07 (0.04, 0.11) | 0.06 (0.04, 0.09) | South |
| Vuolgamjaure | 0.07 (0.05, 0.1) | 0.11 (0.08, 0.16) | North |
| Gosjön | 0.07 (0.05, 0.11) | 0.08 (0.05, 0.11) | North |
| Övre Skärsjön | 0.07 (0.05, 0.11) | 0.16 (0.11, 0.25) | North |
| Brunnsjön | 0.08 (0.05, 0.11) | 0.1 (0.06, 0.14) | South |
| Stora Envättern | 0.08 (0.05, 0.11) | 0.09 (0.06, 0.12) | South |
| Pahajärvi | 0.08 (0.05, 0.12) | 0.08 (0.05, 0.11) | North |
| Ekholmssjön | 0.08 (0.05, 0.12) | 0.06 (0.04, 0.09) | South |
| Hjärtsjön | 0.08 (0.05, 0.12) | 0.11 (0.07, 0.16) | South |
| Tomeshultagölen | 0.08 (0.05, 0.12) | 0.05 (0.03, 0.13) | South |
| Gåtejaure | 0.08 (0.05, 0.13) | 0.21 (0.13, 0.32) | North |
| Valasjön | 0.08 (0.06, 0.12) | 0.26 (0.18, 0.38) | North |
| Mäsen | 0.08 (0.06, 0.13) | 0.05 (0.03, 0.07) | North |
| Stora Skärsjön | 0.09 (0.05, 0.14) | 0.15 (0.08, 0.24) | South |
| Brännträsket | 0.09 (0.06, 0.12) | 0.04 (0.03, 0.06) | North |
| Rundbosjön | 0.09 (0.06, 0.13) | 0.06 (0.04, 0.09) | South |
| Sännen | 0.09 (0.06, 0.13) | 0.23 (0.16, 0.34) | South |
| Dunnervattnet | 0.09 (0.06, 0.14) | 0.17 (0.11, 0.24) | North |
| Njalakjaure | 0.1 (0.06, 0.14) | 0.31 (0.21, 0.44) | North |
| Krageholmssjön | 0.1 (0.06, 0.14) | 0.17 (0.11, 0.25) | South |
| Översjön | 0.1 (0.06, 0.15) | 0.12 (0.08, 0.17) | North |
| Sangen | 0.1 (0.06, 0.15) | 0.1 (0.06, 0.15) | North |
| Älgsjön | 0.1 (0.06, 0.16) | 0.07 (0.04, 0.13) | South |
| Bjännsjön | 0.1 (0.07, 0.14) | 0.09 (0.06, 0.12) | North |
| V. Rännöbodsjön | 0.1 (0.07, 0.15) | 0.08 (0.05, 0.12) | North |
| Djupa Holmsjön | 0.1 (0.07, 0.15) | 0.09 (0.06, 0.13) | South |
| Hökesjön | 0.1 (0.07, 0.15) | 0.07 (0.05, 0.11) | South |
| Öjsjön | 0.1 (0.07, 0.16) | 0.05 (0.03, 0.08) | South |
| Svartsjön | 0.11 (0.06, 0.19) | 0.06 (0.03, 0.1) | South |
| Svartvattnet | 0.11 (0.06, 0.2) | 0.09 (0.05, 0.15) | North |
| Harasjön | 0.11 (0.07, 0.15) | 0.07 (0.05, 0.11) | South |
| St Skärsjön | 0.11 (0.07, 0.16) | 0.06 (0.04, 0.1) | South |
| Tångerdasjön | 0.11 (0.07, 0.16) | 0.06 (0.04, 0.2) | South |
| Bergträsket | 0.12 (0.06, 0.19) | 0.04 (0.02, 0.07) | North |
| Glimmingen | 0.12 (0.07, 0.19) | 0.15 (0.09, 0.23) | South |
| Storasjö | 0.12 (0.08, 0.17) | 0.11 (0.07, 0.15) | South |
| Havgårdssjön | 0.12 (0.08, 0.18) | 0.22 (0.15, 0.33) | South |
| N. Yngern | 0.12 (0.08, 0.18) | 0.13 (0.09, 0.2) | South |
| Stora Tresticklan | 0.13 (0.08, 0.21) | 0.11 (0.07, 0.18) | South |
| Alsjön | 0.13 (0.09, 0.18) | 0.09 (0.06, 0.13) | South |
| Tängersjö | 0.13 (0.09, 0.19) | 0.08 (0.05, 0.13) | South |
| Sidensjön | 0.14 (0.08, 0.25) | 0.05 (0.03, 0.09) | North |
| Hinnasjön | 0.14 (0.09, 0.19) | 0.04 (0.02, 0.06) | South |
| Siggeforasjön | 0.14 (0.09, 0.2) | 0.02 (0.01, 0.04) | South |
| Jutsajaure | 0.14 (0.1, 0.21) | 0.07 (0.05, 0.11) | North |
| Bäen | 0.14 (0.1, 0.21) | 0.06 (0.04, 0.09) | South |
| Fysingen | 0.14 (0.1, 0.21) | 0.06 (0.04, 0.09) | South |
| Horsan | 0.15 (0.08, 0.26) | 0.06 (0.03, 0.1) | South |
| Lilla Öresjön | 0.15 (0.1, 0.22) | 0.1 (0.07, 0.15) | South |
| Tärnan | 0.15 (0.1, 0.22) | 0.05 (0.03, 0.22) | South |
| Degervattnet | 0.15 (0.11, 0.22) | 0.11 (0.07, 0.16) | North |
| Allgjuttern | 0.16 (0.11, 0.23) | 0.15 (0.1, 0.22) | South |
| Svinarydsjön | 0.16 (0.11, 0.24) | 0.18 (0.12, 0.26) | South |
| Bysjön | 0.17 (0.12, 0.24) | 0.05 (0.03, 0.08) | South |
| Älgarydssjön | 0.18 (0.12, 0.26) | 0.13 (0.09, 0.2) | South |
| Överudssjön | 0.18 (0.13, 0.27) | 0.3 (0.2, 0.45) | South |
| St. Lummersjön | 0.19 (0.12, 0.28) | 0.15 (0.1, 0.22) | South |
| Ulvsjön | 0.19 (0.13, 0.27) | 0.11 (0.07, 0.17) | North |
| Hagasjön | 0.19 (0.13, 0.27) | 0.09 (0.06, 0.13) | South |
| Lillsjön | 0.19 (0.13, 0.27) | 0.07 (0.05, 0.1) | South |
| Örsjön2 | 0.19 (0.13, 0.27) | 0.12 (0.08, 0.19) | South |
| Fyrsjön | 0.19 (0.13, 0.29) | 0.22 (0.14, 0.32) | North |
| Ymsen | 0.2 (0.13, 0.3) | 0.88 (0.56, 0.08) | South |
| Fjärasjö | 0.21 (0.14, 0.31) | 0.09 (0.06, 0.13) | South |
| Latnjajaure | 0.22 (0.14, 0.35) | 0.25 (0.16, 0.4) | North |
| Västra Solsjön | 0.22 (0.15, 0.32) | 0.19 (0.13, 0.09) | South |
| Humsjön | 0.23 (0.16, 0.34) | 0.09 (0.06, 0.14) | South |
| Övre Fjätsjön | 0.24 (0.16, 0.35) | 0.28 (0.19, 0.4) | North |
| Remmarsjön | 0.24 (0.17, 0.35) | 0.14 (0.09, 0.2) | North |
| Bästeträsk | 0.25 (0.17, 0.35) | 0.05 (0.03, 0.08) | South |
| Stor-Backsjön | 0.26 (0.17, 0.38) | 0.24 (0.16, 0.35) | North |
| Tronntjärnarna | 0.27 (0.16, 0.44) | 0.15 (0.08, 0.27) | North |
| Stor-Björsjön | 0.29 (0.2, 0.42) | 0.35 (0.24, 0.51) | North |
| Granvattnet | 0.33 (0.22, 0.49) | 0.05 (0.03, 0.08) | South |
